# Splenogonadal fusion: a case report

**DOI:** 10.1186/s13256-023-04241-0

**Published:** 2023-12-15

**Authors:** Ahmed Oshiba, Dina Abdallah, Marwa Abdelaziz, Amir Ibrahim, Mostafa Kotb, Samar Eshiba, Maryam Rizvi

**Affiliations:** 1 Faculty of Medicine, University of Alexandria, Alexandria, 21311 Egypt; 2 Faculty of Life Sciences and Medicine, Guy’s, King’s and St Thomas’ (GKT) School of Medical Education, King’s College London, London, UK

**Keywords:** Accessory spleen, Splenogonadal fusion, Testicular mass

## Abstract

**Background:**

Accessory splenic tissue is a commonly encountered phenomenon in medical literature. Typically, these accessory spleens are found in close proximity to the main spleen, either in the hilum or within the surrounding ligaments. Nevertheless, it is noteworthy that they can also be located in unusual sites such as the jejunum wall, mesentery, pelvis, and, exceptionally rarely, the scrotum. The first documented case of accessory splenic tissue in the scrotum was reported by Sneath in 1913 and is associated with a rare congenital anomaly called splenogonadal fusion. This report describes an infant who presented with a scrotal mass noted by his mother and after examination, investigations, and surgical exploration, it was revealed to be splenogonadal fusion.

**Case description:**

An 8-month-old Caucasian male patient presented with a mass in the left testicle and bluish discoloration of the scrotum, which had been incidentally noticed in the previous 2 months. The general physical examination was unremarkable. Other than a palpable scrotal mass that was related to the upper pole of the testis, the rest of examination was unremarkable. Imaging revealed that this mass originated from the tail of the epididymis without infiltrating the testis and tumor markers were normal. On inguinal exploration, a reddish brown 2 × 2 cm mass was found attached to the upper pole and was completely excised without causing any harm to the testis, vessels, or epididymis. Histopathological evaluation confirmed the presence of intratesticular ectopic splenic tissue.

**Conclusion:**

Although uncommon, splenogonadal fusion can be included in the differential diagnosis of a testicular swelling. Accurate diagnosis allows for appropriate treatment planning which helps to avoid unnecessary radical orchiectomy, which can have a significant impact on the patient’s reproductive and psychological wellbeing.

## Introduction

Accessory splenic tissue is a commonly encountered phenomenon in medical literature. According to Lubarsch, the presence of accessory spleens should be acknowledged as part of the normal adult anatomy due to their frequent occurrence in routine biopsies, with incidences ranging from 2% to 35% [1]. Typically, these accessory spleens are found in close proximity to the main spleen, either in the hilum or within the surrounding ligaments. However, it is noteworthy that they can also be located in unusual sites such as the jejunum wall, mesentery, pelvis, and exceptionally rarely, the scrotum [2].

The first documented case of accessory splenic tissue in the scrotum was reported by Sneath in 1913 and is associated with a rare congenital anomaly called splenogonadal fusion (SGF). This anomaly involves an abnormal attachment of the spleen to a gonad [3]. In the continuous type of SGF, a cord connects the ectopic spleen. This cord can consist purely of splenic tissue, have multiple splenic nodules, or be composed of fibrous tissue. It can take either a retroperitoneal or transperitoneal route and potentially leads to small-bowel obstruction by exerting pressure on it [4]. The discontinuous type involves gonadal fusion with either an accessory spleen or ectopic splenic tissue.

Le Roux and Heddle expressed the view that the latter type was merely a rare variation of an accessory spleen [5]. They also suggested that there was insufficient evidence to support its association with the same etiology or congenital anomalies as the continuous type.

Diagnosis typically occurs when the condition manifests as a testicular mass or is incidentally discovered during ultrasonography, orchiopexy, or inguinal hernioplasty. Having knowledge of this clinical condition can help to avoid unnecessary orchiectomies by enabling the separation and preservation of the testis while excising the lesion.

While SGF typically manifests in childhood, there have been reported cases in adults. Preoperative diagnosis of this condition can be challenging due to inconclusive imaging results, leading to several reported cases being managed with radical orchiectomy. It mimics certain pathologies, making the differential diagnosis clinically relevant.

Here, we present a case of SGF resembling a testicular tumor. Treatment approaches vary from no intervention required to extensive surgeries, underscoring the importance of considering the possibility of an accessory spleen, even if solely to rule out serious pathology.

## Case report

An 8-month-old Caucasian male patient visited the pediatric surgery outpatient department with a complaint of a mass in the left testicle and bluish discoloration of the scrotum, which had been incidentally noticed in the previous two months.

The general physical examination was unremarkable. Upon local examination, a palpable mass was detected in the left scrotum, attached to the upper pole of the testis, with a normal texture. Routine blood tests, renal function tests, and liver function tests yielded normal results. Tumor marker levels [alpha-fetoprotein (αFP) and human chorionic gonadotropin (βHCG)] and lactate dehydrogenase (LDH) were within the normal range.

**Fig. 1:**
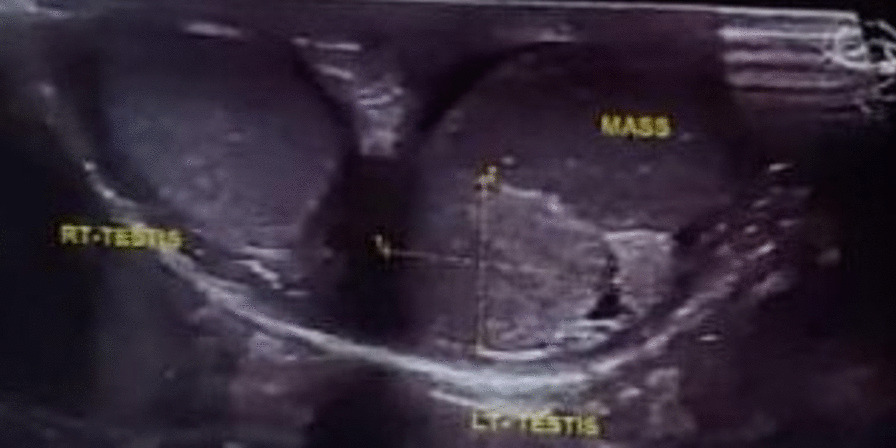
Ultrasound revealed hyper vascular mass lesion related to left epididymal head

**Fig. 2:**
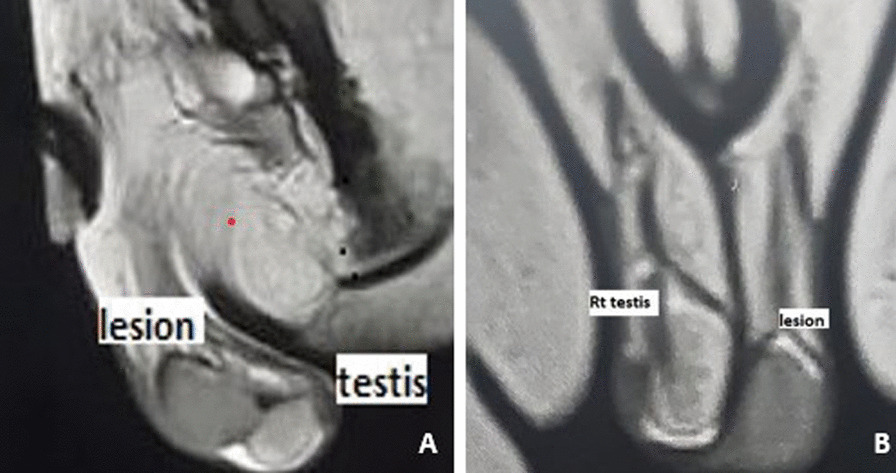
Magnetic resonance imaging. **A** Sagittal and (**B**) coronal T2 sequence revealed well defined extratesticular hypointense mass lesion

Abdominal ultrasonography revealed normal liver, spleen, gallbladder, and kidneys. Scrotal sonography identified a well-defined hypoechoic lesion measuring approximately 2 × 1.3 cm (Fig. 1). Subsequent magnetic resonance imaging (MRI) of the scrotum confirmed the presence of a well-defined mass, approximately 2 cm in size, within the left testis. The mass showed isointensity on T1-weighted images and slight hypointensity on T2-weighted images. It originated from the tail of the epididymis without infiltrating the testis, and no enlarged lymph nodes were observed. Contrast study revealed good enhancement, suggesting a neoplastic etiology such as fibrous pseudotumor or adenomatoid tumor of the epididymis (Fig. 2).

**Fig. 3:**
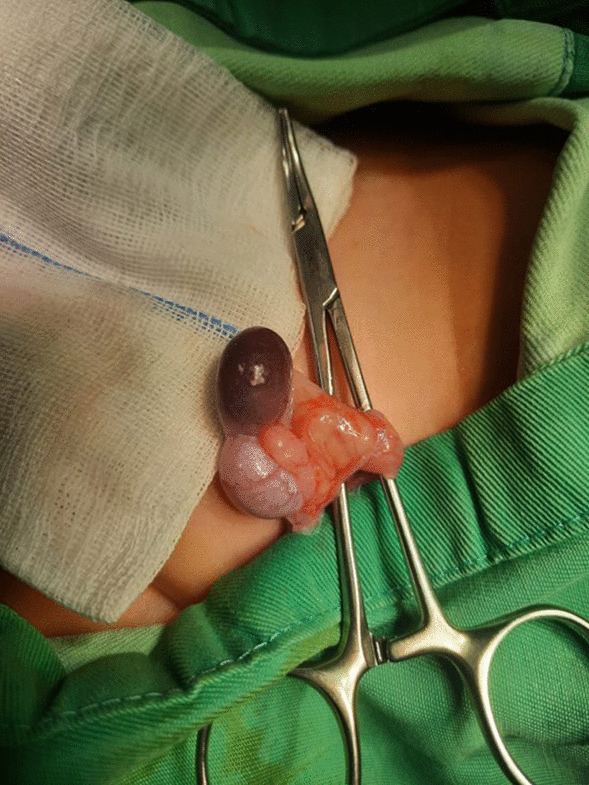
A rounded mass, measuring 2 × 2 cm and reddish-brown in color found at the lower pole of the testis

**Fig. 4:**
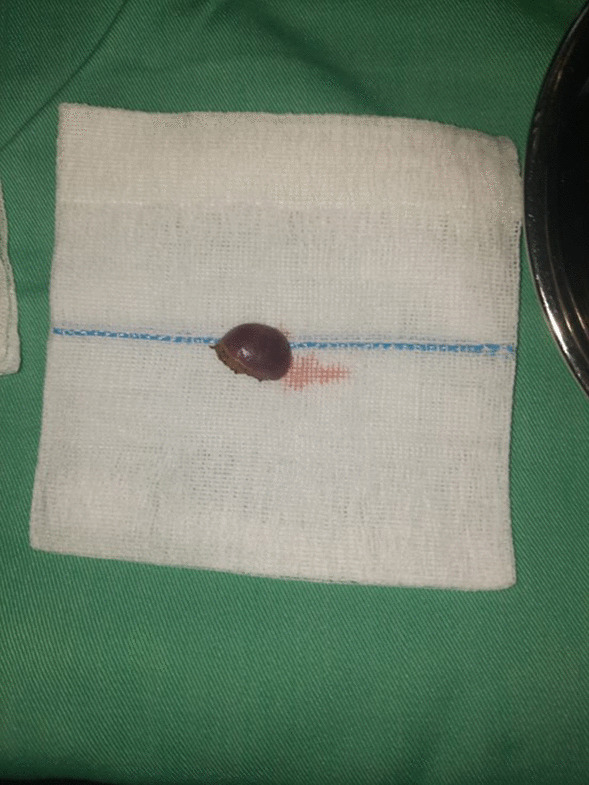
The mass was excised easily without jeopardizing the testis, vessels, and the epididymis

**Fig. 5:**
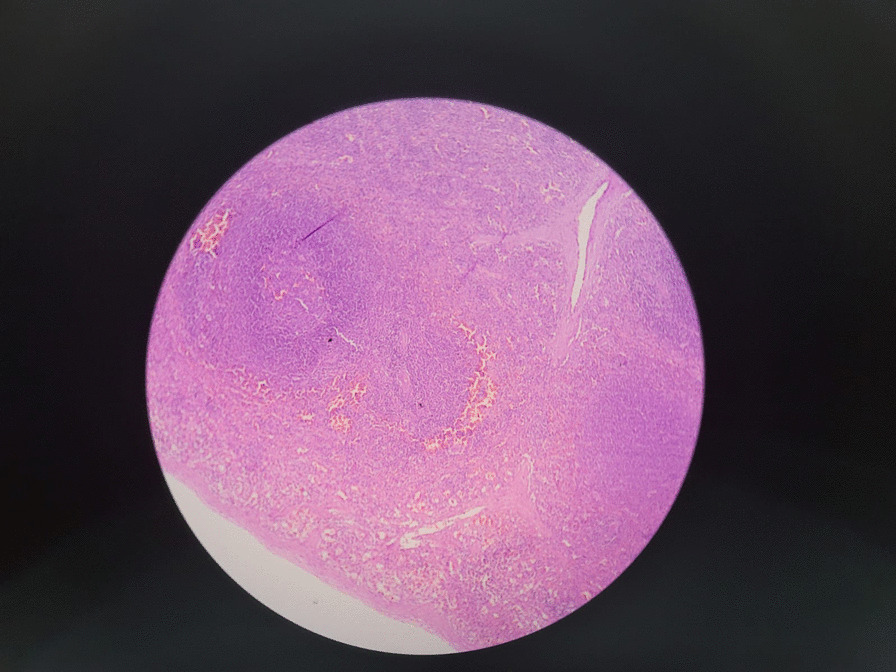
A photomicrograph showing encapsulated splenic tissue composed of reactive lymphoid follicle surrounded by dilated congested sinuses containing red blood cells

Based on these diagnostic findings, the patient underwent left inguinal exploration. The mass, measuring 2 × 2 cm and reddish-brown in color (Fig. 3), was completely excised without causing any harm to the testis, vessels, or epididymis (Fig. 4). The specimen was sent for histopathological examination, which confirmed the presence of intratesticular ectopic splenic tissue (Fig. 5). The patient recovered well during the postoperative period and was discharged on the same day. Subsequent follow-up visits indicated a satisfactory recovery.

## Discussion

Splenogonadal fusion (SGF) is a rare, benign, congenital abnormality characterized by ectopic splenic tissue found in the scrotum [6]. The first reported case of accessory splenic tissue in the scrotum was documented by Sneath in 1913, and, since then, only a limited number of cases have been reported in the medical literature [3]. While the continuous type of SGF involving a cord connecting the ectopic spleen is well-documented, the discontinuous type, which involves gonadal fusion with accessory spleens or ectopic splenic tissue, is considered a rare variation of an accessory spleen [5]. In this case report, we presented a case of SGF in an 8-month-old male patient who presented with a testicular mass and bluish discoloration of the scrotum.

The diagnosis of SGF can be challenging, as it often mimics other testicular pathologies [7]. Imaging techniques play a crucial role in diagnosis and preoperative evaluation. In our case, scrotal ultrasonography initially identified a well-defined hypoechoic lesion within the left testis. This finding raised suspicion of a neoplastic etiology, with contrast enhancement further suggesting this notion. However, magnetic resonance imaging (MRI) provided additional insight into the nature and extent of the mass, and findings were consistent with the presence of splenic tissue found on histopathological examination.

Interestingly, some radiologists have suggested that the use of Doppler ultrasonography can possibly aid in the preoperative differentiation of SGF and testicular malignancies by identifying aberrant blood supply to the fused tissue [8]. It is important to ensure that findings from Doppler ultrasonography are also correlated with clinical data, physical examination, and other diagnostic tests to establish an accurate diagnosis.

The surgical approach for SGF can vary depending on the location of the ectopic splenic tissue and the involvement of adjacent structures, as well as the presence of symptoms. In asymptomatic cases or those in which the size of the mass is small and unlikely to cause complications, a conservative approach with close monitoring may be appropriate. In our case, due to the size of the mass and the presence of symptoms, the patient underwent left inguinal exploration, and the mass was completely excised without causing harm to the testis, vessels, or epididymis.

It is worth mentioning that there is no standardized approach to the surgical management of SGF due to its rarity and limited number of reported cases. Treatment decisions are often made on a case-by-case basis, taking into consideration factors such as the patient’s age, symptoms, tumor characteristics, and surgical expertise. Several studies have used an orchidopexy approach to manage SGF, and Chen *et al*. have described a two-stage laparoscopic staged Fowler–Stephen orchiopexy and suggest it as a surgical option to be performed in cases where routine single-stage orchiopexy is not feasible [9]. The preservation of testicular function is a primary goal in these surgical interventions.

Broadly speaking, the rarity of occurrence of SGF is a major contributing factor to the lack of awareness surrounding the case. An understanding of SGF is of utmost importance due to its potential to mimic other testicular pathologies and the implications it has on patient management. Accurate diagnosis allows for appropriate treatment planning which helps to avoid unnecessary radical orchiectomy, which can have a significant impact on the patient’s reproductive and psychological wellbeing. Continued research efforts are necessary to improve understanding of this rare condition, as well as to establish standardized diagnostic and treatment protocols.

## Conclusion

Accessory splenic tissue in the scrotum is an important consideration when evaluating testicular masses. Its presentation as an asymptomatic testicular mass can easily be misdiagnosed as malignancy, despite its normal characteristics indicating its benign nature. Preoperative laparoscopic assessment can prevent unnecessary radical orchidectomy. However, in cases where salvage is performed for cryptorchidism, surveillance is advised to detect potential future malignancies. Increased awareness of this condition facilitates prompt diagnosis, reducing the likelihood of unnecessary orchiectomies.

## Data Availability

The datasets used and/or analyzed during the current study are available from the corresponding author on reasonable request.
